# The Transformative Role of Artificial Intelligence in Dentistry: A Comprehensive Overview. Part 1: Fundamentals of AI, and its Contemporary Applications in Dentistry

**DOI:** 10.1016/j.identj.2025.02.005

**Published:** 2025-03-11

**Authors:** Lakshman Samaranayake, Nozimjon Tuygunov, Falk Schwendicke, Thanaphum Osathanon, Zohaib Khurshid, Shukhrat A. Boymuradov, Arief Cahyanto

**Affiliations:** a Center of Excellence for Dental Stem Cell Biology, Faculty of Dentistry, Chulalongkorn University, Bangkok, Thailand; b Faculty of Dentistry, University of Hong Kong, Sai Ying Pun, Hong Kong; c Dr DY Patil Dental College and Hospital, Dr DY Patil Vidyapeeth, Pimpri, Pune, India; d Faculty of Dentistry, Kimyo International University in Tashkent, Tashkent, Uzbekistan; e Clinic for Conservative Dentistry and Periodontology, University Hospital of the Ludwig-Maximilians-University Munich, Munich, Germany; f Department of Prosthodontics and Dental Implantology, College of Dentistry, King Faisal University, Al-Ahsa, Saudi Arabia; g Department of oral and maxillofacial surgery, Faculty of Dentistry, Tashkent Medical Academy, Tashkent, Uzbekistan; h Department of Restorative Dentistry, College of Dentistry, Ajman University, Ajman, United Arab Emirates; i Centre of Medical and Bio-allied Health Sciences Research, Ajman University, Ajman, United Arab Emirates

**Keywords:** Artificial intelligence, Dentistry, Challenge, Literacy, Oral health, AI,, Petient care

## Abstract

Artificial intelligence (AI) holds immense promise in revolutionising dentistry, spanning, diagnostics, treatment planning and educational realms. This narrative review, in two parts, explores the fundamentals and the multifaceted potential of AI in dentistry. The current article explores the profound impact of AI in dentistry, encompassing diagnostic tools, treatment planning, and patient care. The Part 2 of the article delves into the potential of AI in patient education, ethics and the FDI communique on AI in dentistry. The review begins by elucidating the historical context of AI, outlining its recent widespread use in various sectors, including medicine and dentistry. The narrative delves into the fundamental concepts of AI, which entails developing machines capable of executing tasks that typically necessitate human intellect. In the biomedical realm, AI has evolved from exploring computational models to constructing systems for clinical data processing and interpretation, aiming to enhance medical/dental decision-making. The discussion delves into the pivotal role of AI models in dentistry, such as Large Language Models (LLM), Large Vision Models (LVM), and Multimodality Models (MM), revolutionizing processes from clinical documentation to treatment planning. The narrative extends to the applications of AI in dental specialties such as periodontics, endodontics, oral medicine and pathology, restorative dentistry, prosthodontics, paediatric dentistry, forensic odontology, oral and maxillofacial surgery, orthodontics, and orofacial pain management. AI's role in improving treatment outcomes, diagnostic accuracy, and decision-making processes is evident across these specialties, showcasing its potential in transforming dental care. The review concludes by highlighting the need for continued validation, interdisciplinary collaboration, and regulatory frameworks to ensure the seamless integration of AI into dentistry, paving the way for enhanced patient outcomes and evidence-based practice in the field.

## Introduction

The term artificial intelligence (AI) has been widely used globally for decades. However, the realisation of its unfathomable potential dawned when the generalised use of a large language model (LLM) was first made available to the public in 2022. Since then, AI use has been widespread in various domains, including medicine and dentistry. In essence, it is the concept of developing machines capable of carrying out intellectual tasks typically done by humans. When a machine demonstrates the ability to make informed decisions, it can be referred to as being artificially intelligent.^1^

AI is dedicated to comprehending and constructing intelligent entities, frequently embodied as software programs.^2^ It can be elucidated as a series of operations devised to execute a particular task.^3^ In biomedical sciences, the initial AI endeavours can be viewed as a sequence of efforts to explore, comprehend, and construct computational models mirroring scientific knowledge and problem-solving strategies. These efforts involve developing and evaluating computational systems for clinical data processing and interpretation alongside modelling clinical reasoning. This approach is aimed to surpass the prevalent logical, statistical, and pattern recognition models for medical decision-making that gained popularity from 1950s onward.^4^

Two primary types of AI prevalent today are narrow AI (weak AI) and general AI (strong AI). Narrow AI can be classified into various subclasses, such as machine learning (ML) and expert-based systems.^5^ ML in turn consists of supervised, unsupervised, or semi-supervised learning. Supervised learning employs labelled data for training, which can limit algorithm adaptability and performance. Unsupervised learning processes unlabelled data but requires greater algorithmic complexity. Semi-supervised learning combines a small amount of labelled data with large volumes of unlabelled data for training.^5^ A key subset of ML is deep learning (DL), which utilizes artificial neural networks (NNs) to identify intricate patterns within the processed data. The primary distinction between DL and traditional ML lies not in how they learn but, in their ability, to capture increasingly complex and hierarchical data representations.

Neural networks (NNs), which form the foundation of DL, include artificial neural networks (ANNs), convolutional neural networks (CNNs), and generative adversarial networks (GANs).^5^ ANNs process information in a feedforward manner, from input through hidden layers to output. CNNs specialize in processing structured grid-like data, employing layers of convolutional filters to extract features. GANs, trained through adversarial learning, generate new data resembling their input.^5^

Presently, within the health professional education (HPE) domain, AI predominantly leverages models such as Large Language Models (LLM), Large Vision Models (LVM), Multimodality Models (MM), Reinforcement Learning (RL) Models, and Generative Adversarial Networks (GANs) and many more.^6^^,^^7^ These are briefly described below.

LLM, epitomised by models like the GPT (Generative Pretrained Transformer), has revolutionised natural language processing (NLP) tasks. These models, trained on vast amounts of textual data, exhibit remarkable abilities in understanding, generating, and summarising textual information. In dentistry, LLMs can be harnessed for many tasks ranging from automating clinical documentation to aiding patient communication through chatbots, thus enhancing overall efficiency and patient satisfaction.^6^^,^^8^, ^9^, ^10^

On the other hand, LVM, represented by architectures like Convolutional Neural Networks (CNNs), excel in image and video analysis and interpretation. In dentistry, LVMs can facilitate the interpretation of radiographs, aid in the detection of pathologies, and assist in treatment planning by analysing intraoral and extraoral images. LVMs contribute significantly to diagnostic accuracy and treatment efficacy by providing precise and rapid assessments.^11^^,^^12^ In addition to CNNs, vision transformers (ViTs) are gaining recognition as advanced tools for image and video analysis. Research suggested that transformer-based models could outperform CNN-based algorithms in diagnostic tasks like caries detection in intraoral images, highlighting their potential to further improve diagnostic accuracy and efficiency.^13^

MM enables AI systems to process and interpret information from diverse modalities such as text, images, and voice. These models hold potential in dentistry by facilitating comprehensive patient data analysis, enabling holistic treatment planning, and enhancing interdisciplinary collaboration among dental professionals.^6^

Reinforcement Learning (RL) models are unique in ML, as they learn through trial and error by interacting with their environment.^14^ This approach has evolved into a dynamic and transformative element of AI, enabling intelligent decision-making in complex and ever-changing situations. The distinctive capabilities of RL make it particularly useful in simulation-based learning, such as robotic surgery training and clinical decision-making. These models are powerful tools for developing adaptive and interactive learning environments, especially in areas that require decision-making skills and real-time feedback. In dental education, RL can enhance haptic simulations, virtual reality experiences, and robotic-assisted dental surgeries.^15^^,^^16^

In dentistry, input data encompass visual (eg, clinical images and radiographs), textual (eg, electronic health records), or audio modes. Neural networks (NN) process these inputs to generate various outputs, such as diagnoses, treatment plans, disease predictions, or prognoses. Diagnosis may involve deciphering clinical cues, cephalometric analysis, or lesion identification. AI in dentistry also aids in recognising structures, analysing results, converting speech data, and facilitating data acquisition for computer-aided design/computer-aided manufacturing (CAD/CAM) processes. Additionally, AI may utilise gene analysis, prioritise risk factors, or predict disease outcomes.^17^

**Fig. 1:**
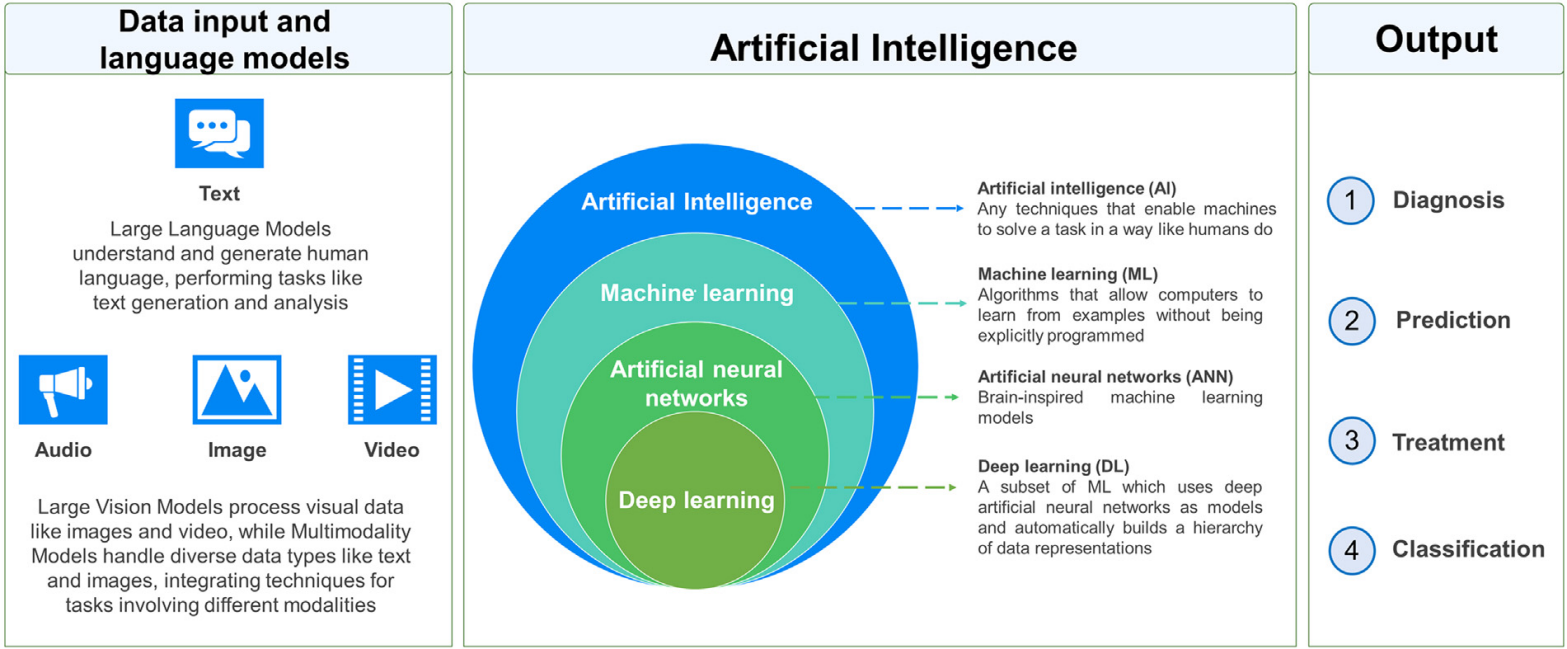
The working of AI in a schematic format.^5^

**Fig. 2:**
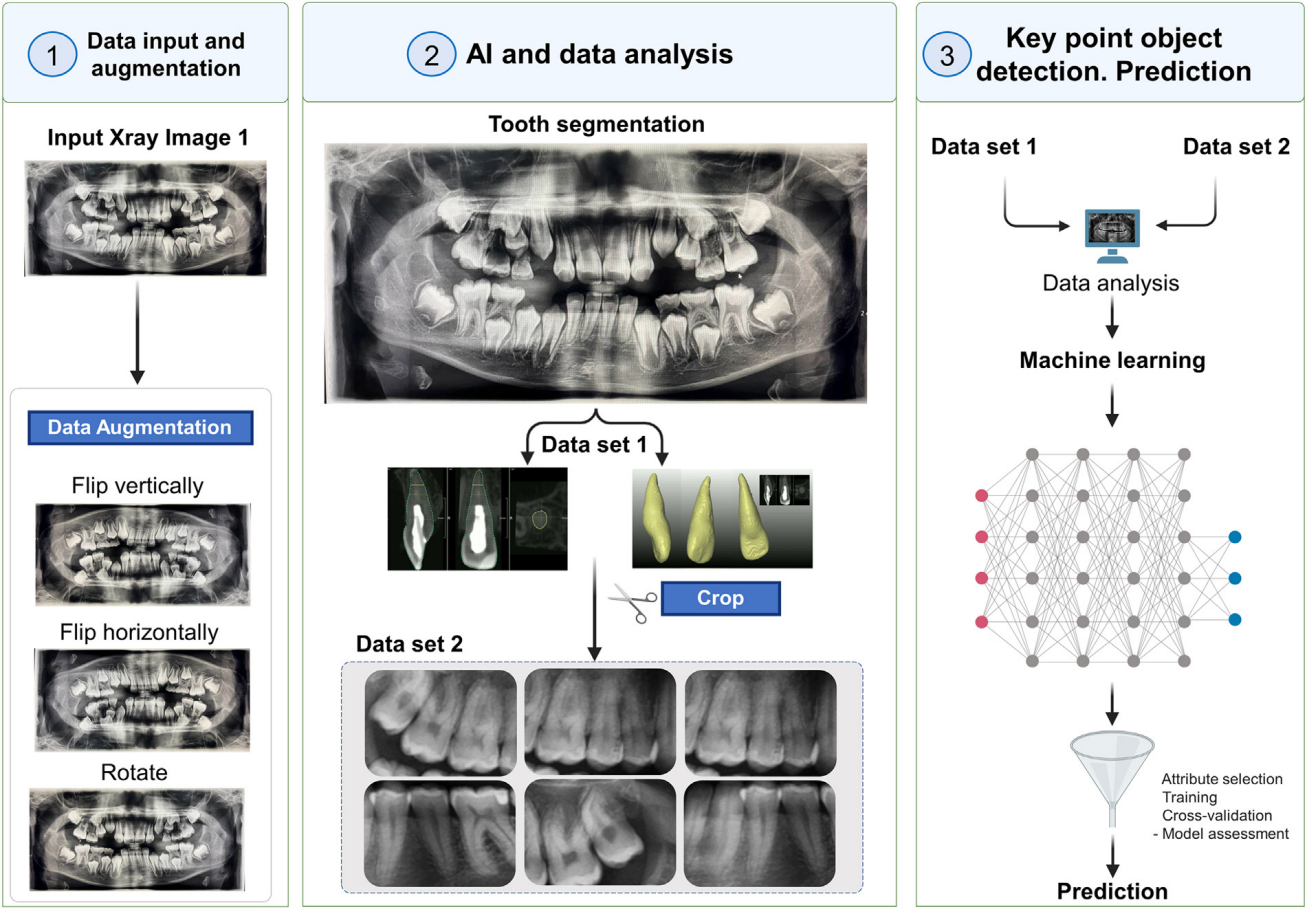
Schematic representation of working AI in Dentistry.^18^

As we explore the role of AI in dentistry, it becomes evident that LLMs, LVMs, and MMs offer unprecedented opportunities for optimising clinical workflows, improving diagnostic accuracy, and ultimately enhancing patient care. However, alongside these opportunities come challenges that need to be addressed. A schematic illustration of the working of AI can be seen in Figures 1 and 2.

Currently there is limited presence of AI software in dentistry. However, progress has been made in areas such as dental image analysis, caries detection, orthodontic treatment planning and analysis, endodontic lesion detections on periapical radiographs, oral pathological lesion detection, and electronic patient record management with the aid of AI.^5^

While existing reviews have examined the landscape of dental AI, this article aims to provide a comprehensive narrative of AI evolution within dentistry. It summarises recent advancements in AI research specific to dentistry and explores the intricate relationship between evidence-based dentistry and AI. Additionally, it addresses the challenges of integrating AI in dentistry, offering a holistic perspective crucial for navigating the future trajectory of AI-driven dentistry.

## Applications of AI in dentistry

We review below the multiple ways in which AI could be applied to almost all facets of dentistry.

### AI in periodontics

Various treatment strategies exist for managing periodontally compromised teeth,^19^ but disease prognosis has been hampered due to poor diagnostics and human error. AI has been explored and advanced in several aspects to enhance the diagnosis, treatment, and monitoring of periodontal care. For instance, CNNs are trained and able to detect chronic gingivitis from clinical intraoral images. ANNs have been experimentally used to assess periodontitis grades in a number of studies. Some models achieve up to 85% accuracy in correctly classifying periodontal patients based on their radiographic bone loss profiles.^20^ Determining bone loss in both panoramic and periapical radiographs has been investigated, facilitating the accurate diagnosis of periodontal bone loss.^21^^,^^22^ A meta-analysis of 10 publications reveals that AI performance demonstrates high mean sensitivity (87%), specificity (76%), and accuracy (84%) in assessing alveolar bone loss and periodontitis from panoramic and periapical radiographs.^23^ Further, the use of AI for detecting furcation involvement in axial CBCT images has been investigated. Results exhibit the Resnet101V2 deep-learning model performs with distinctively high accuracy, precision, and F1 score.^24^ A high correlation has been observed between the AI models and radiological diagnoses. In this regards, a study has revealed an overall whole-jaw Pearson's correlation coefficient of 73% between AI and radiologists and an intraclass correlation value of 91%.^25^

Furthermore, CNNs can be utilised for prediction of periodontal disease. In one study, a CNN exhibited a precision rate of 73.4 and 82.8% for predicting the necessity of tooth extractions for premolars and molars, respectively.^26^ Another study demonstrated that a logistic regression model and a NN for the prediction of tooth loss in periodontitis patients exhibited moderate specificity and high sensitivity and precision in comparison to clinical tooth prognostic systems.^27^ Periodontal disease risk prediction using ML in diabetic patients^28^ found that smoking habits, low educational levels, high income-to-poverty ratio, high albumin levels and high alanine aminotransferase levels were relevant variables for predicting periodontitis.^28^ At molecular levels, ML integrated with Mendelian randomisation and single-cell sequencing data revealed a novel approach to identifying gene interactions in periodontitis,^46^ leading to the potential use of these genes in predictive diagnosis and treatment follow-up for periodontitis patients.^29^

ChatGPT has been explored for its ability to classify periodontal disease status according to the relatively new, 2018 classification. The latter model correctly identified the stage, grade and extent of periodontal disease in one study. This performance required additional fine-tuning to improve the accuracy.^30^ Among four different LLM models (ChatGPT model GPT 4.0, Google Gemini, Google Gemini Advanced, and Microsoft Copilot), ChatGPT 4.0 was superior (>8 out of 10 in scale) in response to open-ended questions in periodontology, confirming its potential in enhancing patient education to be employed as dental professional co-pilots.^31^ LLMs can also be tailored to be focused on periodontology, yielding to higher accuracy rates (upto 81.0%).^32^

ML models effectively identify the correlation between systemic well-being and poor periodontal status.^33^ In this context, localised variations in gingival indices and periodontal diseases were associated with blood pressure, body mass index, and a number of other systemic findings. Interestingly, one models has revealed a link between early periodontal disease, gingivitis, and optic nerve abnormalities.^50^ Additionally, co-occurrence of periodontal diseases has been observed with joint swelling and a family history of eye diseases.^33^ Another AI version, Local interpretable model-agnostic explanations (LIME) identifies key systemic factors contributing to periodontitis, such as arthritis, sleep disorders, hypertension, elevated cholesterol levels, and obesity, aligning with clinically recognised associated conditions.^34^

The foregoing indicates that, AI has significantly advanced periodontics by improving diagnosis, treatment, and monitoring of periodontal diseases. ANNs and CNNs demonstrate accuracy in detecting bone loss and predicting treatment outcomes and disease progression, while generative AI can be utilised to enhance patient education and professional support. These innovations highlight AI's transformative potential for precision diagnostics, personalised care, and interdisciplinary integration appertaining to periodontology.

### AI in endodontics

Early detection of periapical lesions due to pulp infections is vital to improve treatment outcomes, prevent spread of infection, and mitigate related adverse consequences.^35^^,^^36^ It is known that clinician visualisation, of CBCT scans demonstrate superior accuracy in identifying periapical lesions compared to conventional and digital periapical radiographs.^37^ However AI technology appears to improve the accuracy of detecting pathology in CBCT images. In one study, DL demonstrated 93% detection accuracy with high specificity, positive predictive value (PPV), and negative predictive value (NPV).^36^ A CNN system achieved 92.8% reliability in correctly identifying periapical lesions on CBCT images.^38^ In panoramic radiographs, a DL algorithm detected periapical pathoses at 60% precision and 58% F1 score compared to evaluations of experienced oral and maxillofacial surgeons.^39^ A deep CNN model based on the UNET algorithm achieved sensitivity, precision, and F1 scores of 92%, 84%, and 88%, respectively.^40^ Another study utilised a similar algorithm but resulted in lower accuracy.^41^ A modified DL model achieved an 82% F1 score in detecting periapical periodontitis using periapical radiographs, indicating potential for improved diagnostic accuracy,^42^ leading to the improvement of dental professionals' ability to detect apical radiolucencies on intraoral periapical radiographs.^43^ A recent systematic review concluded that DL algorithms are more effective than expert clinicians in accurately detecting periapical radiolucent lesions in dental radiographs.^44^ All these studies indicate the superiority of AI in detecting apical pathology and is feasible for future endodontic practice.

AI-driven dental pulp cavity segmentation in CBCT images is also another application of AI to facilitate endodontic treatment. Automated pulp cavity segmentation assists in endodontic workflow and minimally invasive endodontic procedures, and reduces the time needed compared with manual segmentation.^45^ It has also been shown that CNN models (ResNet-101 and - 50, DenseNet-121 and - 161, and Inception-V3) are able to detect C-shape canals in panoramic radiographs.^46^ These models outperform dental practitioners in identifying C-shaped canals in mandibular second molars. Additionally, AI has been explored to diagnose the status of root canal fillings visualised in CBCT images, including the adequacy of obturation, overfilling, short filling and voids in fillings. Results indicate very high accuracy in detecting these parameters (over 80%).^37^ Further, ANNs can be employed to determine the working length from a radiograph. ANNs determined the correct position of a file in a canal more precisely (96%) than endodontists (76%).^47^ LLMs have also been explored for pulpal and periradicular diagnosis. Bing and ChatGPT 4.0 outperformed ChatGPT 3.5 and Bard in the accuracy of diagnosis and treatment recommendations.^48^ In addition, using English language for case information resulted in better performance.^48^ Google Gemini exhibited an accuracy similar to that of endodontists in response to a question related to the management of traumatised permanent teeth.^49^

### AI in oral medicine and pathology

Recent advances in cancer diagnostics have emphasised the virtues of digital image analysis, which entails extracting critical information from images for tasks such as feature delineation (segmentation) or label assignment (classification).^25^^,^^50^ Numerous customised ML methods, leveraging feature analysis, have succeeded in various diagnostic applications by predefining specific features and processing workflows (Figure 1). Such data have been explored with AI models to facilitate oral cancer diagnosis for instance, and these include intraoral images, radiographs, and other diagnostic data. AI is also employed to facilitate pathologists tasks in order to assist histolopathological diagnoses derived from tissue sections. High-quality data from these technologies would will be amenable for evaluation by ML methods in the future, enabling swift, precise, and accurate diagnosis of head and neck tumours, thereby paving the way for improved prognosis. Below are some AI interventions that could enhance the quality of life for head and neck cancer patients.

**Fig. 3:**
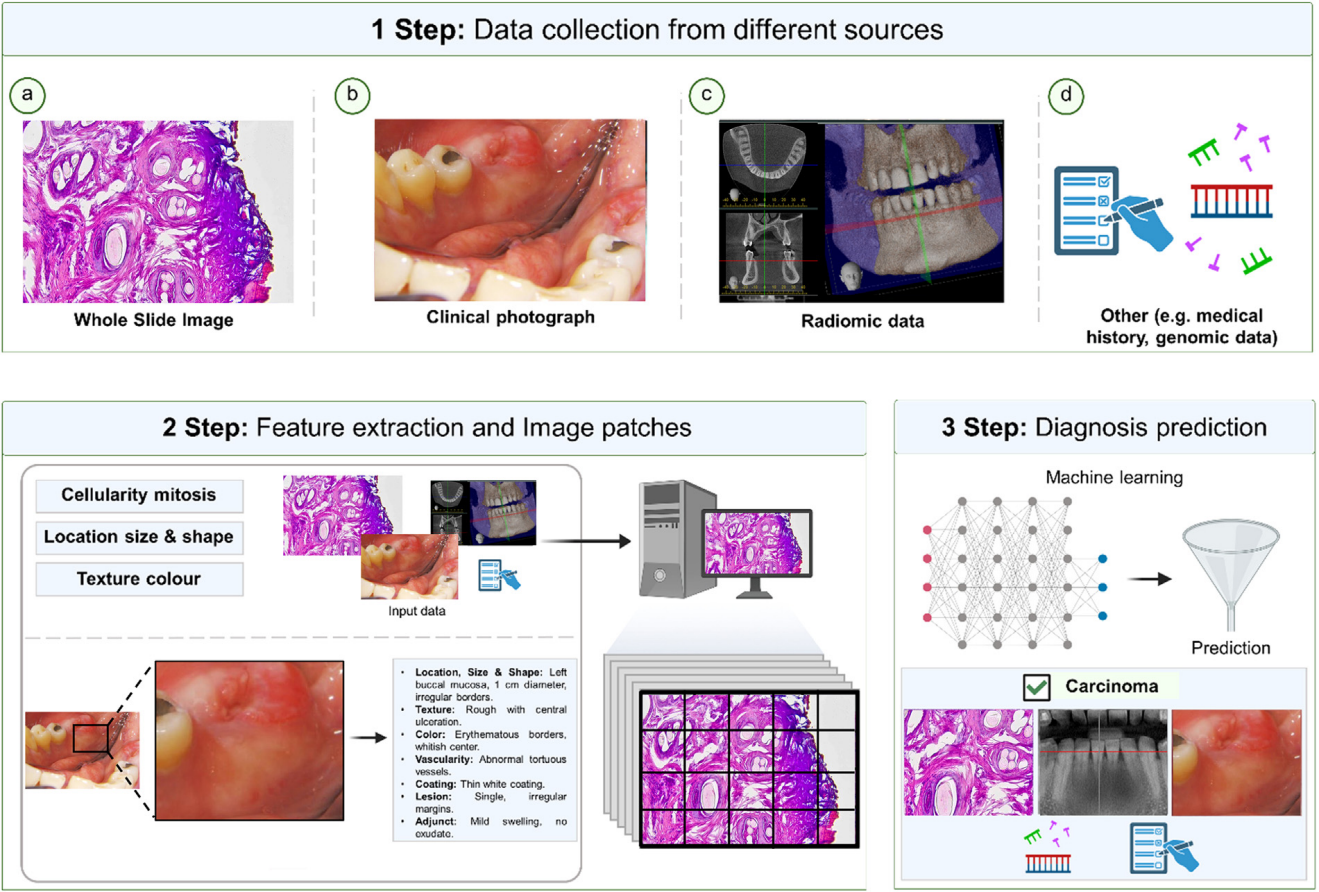
A schematic of feature extraction from primary diagnostic imaging modalities to aid outcome prediction. Images are reproduced from Charoenlarp et al.,^59^ under the terms of the Creative Commons Attribution Noncommercial License (http://creativecommons.org/licenses/by-nc/3.0).

Clinical data from intraoral or endoscopic images have been utilised to train AI programmes for detecting oral cancers,^51^ nasopharyngeal cancers.^52^ oropharyngeal cancers,^53^ laryngeal cancers,^54^ and miscellaneous oral mucosal lesions.^55^ ML models for instance were able to differentiate normal laryngeal tissues from malignant tissues in endoscopic images using textural information.^54^ Hence, incorporating AI technology with an endoscopic system could enhance the early detection of head and neck cancer lesions. With such technology, the detection of oral cancer in clinical oral images can be improved. ML classification models from multispectral narrow-band imaging outperformed those from white-light endoscopy in distinguishing oropharyngeal squamous cell carcinoma from healthy mucosa, with improved accuracy linked to angiogenic and inflammatory surface changes.^53^ Further, CNN models are able to classify oral cancer using fluorescence visualisation. Results indicate that precision was 80.3%, 82.1%, 84.2%, and 94.1% for oral cancer, oral potentially malignant disorders, benign disease, and normal mucosa, respectively.^56^ With a smartphone-based intraoral dual-modality imaging platform, CNN models were trained to distinguish oral premalignant and malignant lesions with an accuracy of more than 86 %.^57^ CNN models exhibit very high precision (100%), specificity (99%), and accuracy (97.5%) in detecting oral cancer from digital images.^58^ However, distinct models specifically perform particular tasks. In this regards, DenseNet121, VGG19, and EfficientNet-B0 are excellent for binary classification^58^ while efficientNet-B4, Inception-V4, and Faster R-CNN excel in multiclass classification and object detection.^58^ A schematic of extraction and enalysis of key features from primary diagnostic imaging modalities for predicting clinical outcomes is shown in Figure 3.

AI technologies are increasingly utilised to analyse a variety of radiological and special technique-derived images depicting head and neck cancer and other soft and hard tissue abnormalities. CT-based textural analysis has been utilised to assess malignancies in the head and neck region.^60^ The spectral dual-energy computed tomography data obtained from multienergy virtual monochromatic image datasets is used to systematically classify Warthin tumour and pleomorphic adenoma with a remarkable accuracy of 92%.^60^ An automated algorithm has been formulated to identify nasopharyngeal squamous cell carcinoma on PET/CT imaging, resulting in a remarkable accuracy of 100% in identifying hypermetabolic lesions exceeding 1 cm in size.^61^ A systematic review of publications related to AI for the detection of head and neck cancer using CT, PET, MRI, Planar scans, or panoramic radiographs demonstrated a wide range of accuracy (82.6%-100%), sensitivity (74%-99.68%) and specificity (66.6%-90.1%).^62^ Hence, AI integration in radiograph data could enhance the accuracy of detecting head and neck cancer.^62^ On the contrary, the usefulness of ML in detecting intraosseous lesions in jaw bones has yet to be determined, as a systematic review shows a moderate mean F1 score of 0.71, accuracy of 0.86, sensitivity of 0.82, specificity of 0.88, and precision of 0.67.^63^

Numerous studies have utilised whole-slide imaging of stained, histopathological slides to develop algorithms for evaluating oral squamous cell carcinoma (OSCC),^60^ oral potentially malignant disorders (OPMD),^64^ laryngeal squamous cell carcinoma (SCC),^65^ oropharyngeal SCC,^66^ and multiple HNC sites.^67^ In these studies, various ML approaches were employed to delineate specific histological features and conduct downstream statistical analysis, aiming to differentiate between benign and malignant lesions based on differences in spatial architectural patterns.^64^^,^^68^ Unsupervised ML techniques have been utilised to investigate the capacity for identifying tissue compartments in oropharyngeal squamous cell carcinoma and tissue microarrays where morphometric classification of epithelial and stromal tissues achieved a pixel-level F1 score ranging from 80% to 81%.^66^ DL algorithms for stimulated Raman scattering histology demonstrated significant potential in diagnosing laryngeal squamous cell carcinoma, achieving an accuracy of 90%.^65^ This method effectively identified tissue neoplasia at simulated resection margins, enhancing the delineation of the borders between the healthy and tumour tissues, assisting precise surgical resection and reducing disease recurrence potential.

### AI in restorative dentistry and prosthodontics

AI technology has potential applications in restorative dentistry for various diagnostic functions and treatment approaches, including the detection of vertical root fractures,^69^ apical lesions,^36^ overhanging restorative materials,^70^ tooth wear evaluation,^71^ tooth shade matching, identifying dental caries,^72^^,^^73^ and classifying of edentulous areas.^74^ These applications offer promising prospects for improving clinical practice and enhancing patient care.

As described above, numerous studies have investigated the utilisation of DL systems in various oral and maxillofacial imaging procedures, including periapical radiography,^72^ panoramic radiography,^75^ and CT images.^76^ Object detection functionality has been applied to diagnose abnormalities or anatomical structures in panoramic images.^76^

In addition to the above, AI has been used in restorative dentistry to predict the longevity of CAD/CAM restorations,^77^ the prediction and precision of colour matching of artificial teeth.^78^ AI has also been utilised in smile analysis in the realm of aesthetic dentistry.^79^ Smile design, both in 2D and 3D, is employed mainly in patient communication and also planning for restoration. However, one study has shown that the participants’ preference for smile design was inclined towards a manual design over a design generated by AI.^80^

The treatment process for preparing a dental crown typically involves tooth preparation, impression taking, cast trimming, restoration design, fabrication, try-in, and cementation. While CAD/CAM systems have revolutionised the design work, commercial systems primarily relied on pre-existing tooth libraries for crown design, lacking customisation for individual patients.^77^ Recently, AI has been applied to address this limitation. Hwang et al.^81^ and Tian et al.^82^ introduced novel approaches based on 2D-GAN models to generate customised crowns by learning from technicians’ designs, using 2D depth maps derived from 3D tooth models as training data. Additionally, Ding^83^ utilised a 3DDCGAN network for crown generation, employing 3D data directly and producing crowns with morphology resembling natural teeth. Integrating AI with CAD/CAM or 3D/4D printing offers a more efficient workflow. Several workers have investigated the use of generative AI for dental prosthesis design.^84^ Integrating AI in design software exhibited a superior outcome than using conventional software devoid of AI. However, compared with experienced humans, knowledge-based AI is still ineffective for crown design, especially with regards to their anatomy and morphology. While generative AI can enhance the efficiency of dental practices and improve patient outcomes, its integration into dental workflows must be approached with caution, ensuring rigorous testing and continuous oversight.

Furthermore, AI has been used for shade matching^85^ and predicting the debonding of CAD/CAM restorations. A recent scoping review by Kong and Kim focused on the use of AI to enhance the efficiency and precision of dental crown finish lines, colour matching, and prediction of debonding probability.^86^ Ali et al. presented an advanced approach for automated detection and enumeration of teeth and dental prostheses in panoramic X-rays using a dual CNN system combined with an optimisation algorithm.^87^ The proposed method integrates separate YOLOv7 models for teeth and prosthesis detection, addressing the limitations of existing systems that fail to consider complex dental restorations such as implants, crowns, and bridges.

This novel integration of prosthesis data into the tooth numbering process resulted in high precision and recall values, demonstrating its potential to automate dental charting and improve clinical record-keeping accuracy in prosthodontics.

### AI in paediatric dentistry

The use of AI in pedodontics has thus far focused primarily on detection and prediction models. Numerous ANNs and CNNs have been trained for image analysis to detect and classify supernumerary teeth, mesiodens, early childhood caries, stage of the dentition, pit and fissure sealants, dental caries, dental plaque, and ectopic eruptions.^88^ ML was employed to develop oral health assessment toolkits to predict Children's Oral Health Status Index (COHSI) score and referral for treatment needs.^120^ The latter toolkit had 93% of sensitivity and 49% of specificity.^89^ It can be used to evaluate the treatment need in school-based programs or to compare pre- and postinterventive programs to determine the change in treatment needs. Utilizing an array of variables, including the criteria established by the International Caries Detection and Assessment System (ICDAS) in conjunction with additional clinical factors, ML algorithms like decision trees, random forests, and extreme gradient boosting (XGBoost) were applied alongside logistic regression methodologies for the purpose of caries prediction.^90^ Studies indicate that AI models have the potential to determine the onset of caries development in both primary and permanent dentitions. Notably, clinical factors such as caries experience, the non-utilisation of fluoridated toothpaste, parental education levels, elevated frequencies of sugar intake, and inadequate parental perceptions of their children's oral health emerged as predominant predictors of caries in permanent dentition.^90^ Additionally, a caries risk prediction model for public health purposes have been developed by Qu et al. Using twelve nonbiological questions, this model predicted the onset dental caries in children aged <60 months.^91^

AI-based dental plaque detection could be advantageous for dentists and guardians in monitoring the oral health of children. A CNN-based model demonstrated a superior mean results in comparison to dentists for detecting dental plaque on photographs of the primary dentition.^92^ The model exhibited a performance that is clinically acceptable when compared with an experienced paediatric dentist. With the help of an accessible intraoral camera, parents, in future, should have the ability to capture intraoral images and employ such models for the assessment of residual dental plaque.^92^

Further, AI models demonstrated a superior ability to accurately identify proximal caries in bitewing radiographs in comparison to dentists. One study revealed a high matrix scoring for AI to detect proximal caries. The model achieved the following performance metrics: 88.8 percent sensitivity, 98.8 percent specificity, 95.8 percent precision, 96.4 percent accuracy, and an F1-score of 92 percent, by surface.^93^ Additionally, it has been shown that dentists utilizing AI-assistance exhibit enhanced efficiency in detecting caries from bitewing radiographs.^94^ A systematic review indicates that AI models are clinically acceptable for identifying proximal caries from bitewing radiographs. Additionally, AI-based smartphone applications for caries detection provide ease of home screening for parents thus engaging both children and guardians in pediatric oral health care.^95^

In terms of early childhood caries detection, AI is employed for caries detection, classification, and localization. Such a well developed model was able to diagnose using image analysis, an overall accuracy of 97.2%.^96^ However, the model exhibited less accuracy in cavity segmentation. LLMs were investigated to determine their potential for parent education regarding early childhood caries. These models appropriately responded to the commonly-asked questions formulated by the experts.^97^

One of the major treatment components in paediatric patients is behaviour management.^98^ Integrating AI in behaviour management has been proposed through a variety of approaches. AI-adaptive personalized lessons and gamification was introduced to develop patients’ motivation and engagement in oral health and treatment compliance. AI-based virtual assistance could be beneficial to support explanation and feedback. The latter methodology can attract children to do various tasks so as to distract them from the treatment process. Engaging in interaction via text chatbot or voice conversation could emotionally support patients and alleviate anxiety and stress.

### AI in forensic odontology

Forensic odontology plays a crucial role in identifying victims of mass disasters and in cases involving decomposed, burned, or skeletonized remains. Within this field, age estimation, gender determination, and facial reconstruction are key subdisciplines, particularly valuable when information about the deceased is scarce. AI models for the latter purposes have been developed and utilised in several studies, including human bite mark analysis, predicting mandibular morphology, sex determination, age estimation, and dental comparison.^85^ Recent research has focused on creating automated identification systems using AI algorithms to improve these forensic procedures. Most of the developed models are based on CNNs and ANNs.

In one study, ANN identified on average 82% correct matches between bite marks and the perpetrators. Another model showed superior performance in comparison to support vector regression for mandibular morphology prediction from craniomaxillary variables for the purpose of forensic facial reconstruction.^99^ In regards to sex determination, multiple variables have been introduced and investigated on multiple models, including hyoid bone, mandibular condyles, coronoid process, coronoid height, condyle height, ramus height, sigmoid notch, mandibular canine width, nostril width, and intermolar width.^100^ Notably, a ML model demonstrated a markedly enhanced predictive accuracy, thereby highlighting its potential for sex determination in forensic and anthropological work,

Age estimation is one of the fundamental and key components of forensic odontology. AI can now be employed to determine an individual's age for personal identification. In particular in scenarios where adequate documentation is absent, age estimation becomes essential for delineating the specific legal actions that may be applicable to that individual or to those associated with the individual. Many variables have been employed as parameters for age estimation, including hand-wrist radiographs, panoramic radiographs, 3-dimensional computed tomography.^101^ High accuracy of AI for age estimation was reported when using orthopantomography. Model accuracy was higher than 95%. The mean absolute errors (MAE) associated with age estimation derived from panoramic radiographs exhibited a lower value in the younger age groups whereas higher errors were observed in the older population. In this context, the MAE was 1.94 years for the age cohort of 10-20 years, while a MAE of 13.40 years was observed for the 90-100 years group.^102^

Lastly, personal identification is considered a significant aspect in the field of forensic odontology. The process of correlating premortem and postmortem data from individuals affected by mass casualty events represents a substantial workload. Six CNNs models, VGG16, ResNet50, Inception-v3, InceptionResNet-v2, Xception, and MobileNet-v2, were compared for their sensitivity in personal identification, using orthopantomography.^103^ All model performances demonstrated accuracy of 80% or above.^103^ However, the VGG16 model exhibited the highest accuracy at 100.0%. These preliminary results demonstrate the potential application of AI-assisted personal identification from a large database of orthopantography outcomes. These models have shown encouraging results, ushering in a new realm of AI-driven research in this area. It is highly likely that, in future, AI will play a key role in forensic odontology when the identification of individuals from hard tissues such as teeth and bone is required.

### AI in oral and maxillofacial surgery

AI tools have been developed and explored for various applications in oral and maxillofacial surgery. These range from image analysis, surgical planning, robotic-assisted treatment, and clinical decision-making. One notable use of AI in image analysis is in assessing the impacted mandibular third molars from radiographic images to determine the surgical difficulty in order to design treatment planning.^52^ With AI assistance, less experienced practitioners can achieve diagnosis and classification of the impacted mandibular third molars comparable to that of the experts’.^76^ Automated detection of oral structures in clinical and radiographic images enhanced AI-facilitated complex dataset analysis for optimal treatment strategies.^104^ For example, to assess the condylar seating prior to orthognathic surgery.^105^ Results demonstrated that using AI assistance, 71% of the condylar heads were correctly seated.

With AI-enhanced visualisation, surgeons can precisely evaluate treatment approaches and predicted outcomes in order to adapt and modify the appropriate options for each patient, thus supporting clinical decision-making.^106^ Supervised CNN models were trained in lateral cephalograms and occlusal views of scanned dental models. Data indicate that the trained models exhibited high performance in predicting the surgery-first approach.^107^ Such approaches could facilitate the surgical treatment planning of skeletal Class III patients for example. A study in treatment planning for dental implant placement revealed the alignment between an AI-generated plan and a clinical plan.^108^ Although there were some discrepancies between these two approaches, AI assistance could reduce the planning time employed. Although LLMs may assist clinical decision-making, all tested models (Bard, GPT-3.5, GPT-4, Claude-Instant, and Bing) in a recent study exhibited a low percentage of accuarcy (less than 40%).^109^

Robotic-assisted surgery has been implemented in oral and maxillofacial surgery, especially in dental implant placement. Automated robot-assisted surgery augments the precision and accuracy associated with the placement of dental implants.^110^ In one study the latter system exhibited a high degree of accuracy, evidenced by mean deviations measuring 0.61 mm in the coronal plane, 0.79 mm in the apical plane, and 2.56 degrees in terms of angular deviation, indicating precise positioning.^110^ Using robotic-assisted surgery augmented with AI resulted in lower point errors of bilateral osteotomy planes in a mandibular tumour model study, offering precision and accuracy of such surgery.^111^ Therefore, AI integration into robotic systems could enhance the diagnosis, personalise the surgical plans, as well as improve surgical skills of less experienced surgeons in particular.

### AI in orthodontics

Integrating AI into orthodontics enhance diagnostic efficacy and accuracy, improving treatment planning, decision-making, and patient outcomes. Automated detection of orthodontic landmarks of cephalogram using AI algorithms is now relatively common. In one such study accurate detection within a 2 mm precision threshold was reached for more than 85% of landmarks.^112^ A systematic review revealed the variability in landmark detection accuracy across studies. Reported success rates for orthodontic landmark detection for 1-mm, 2-mm, 2.5-mm, 3-mm, and 4-mm precision thresholds were 65%, 81%, 86%, 91%, and 96%, respectively.^113^ Additionally, CNNs were utilized to analyse intraoral photographs for detecting and classifying crossbite. Results demonstrate excellent potential in clinical image processing for identifying crossbites from noncrossbites at an accuracy of 98.57 % and classifying frontal crossbites from lateral crossbites at an accuracy of 91.43 %.^114^ Further, as AI engages in various steps of orthodontic treatment it can be used in the prediction of treatment planning and outcomes as well as in improving clinical decision-making.^115^ When seven ML models were tested for tooth extraction for orthodontic treatment^116^, the Stacking Classifier model exhibited the highest accuracy and area under the curve (91.2%).

### AI in orofacial pain, temporomandibular disorders, and sleep apnea management

Diagnosis of orofacial pain and temporomandibular disorders is a challenging task. To assist and facilitate such patient care, AI models have been developed and explored. A multilayer perceptron neural network model was developed, and demonstrated significantly higher diagnostic accuracy than evaluation by general dentists, especially in conditions involving nondental and referred orofacial pains.^117^ Further, AI algorithms developed for automated TMD diagnosis can serve as decision-support tools for clinicians. In this regards, a systematic review reports that AI models demonstrated a pooled accuracy of 0.91 and specificity ranging from 73% to 100% for the diagnosis of temporomandibular disorders. However, a high risk of bias in the included 17 studies was apparent.^117^ Random Forest and logistic regression analysis AI models application using brain imaging data were able to classify subtypes of neuropathic facial pain (trigeminal neuralgia and trigeminal neuropathic pain) and healthy controls.^118^ However, the accuracy of this model was only 51%.^118^ An LLM for assisting differential diagnosis of patients with odontogenic pain and temporomandibular disorder based on validated questionnaires have been developed, achieving an accuracy rate of 86%.^119^ The incorporation of advanced algorithms and extensive datasets may further enhance the diagnostic precision of orofacial pain and temporomandibular joint disorders, going forwards.

An automatic prediction model for diagnosing obstructive sleep apnea in temporomandibular disorder patients has been developed, achieving 80%-91% accuracy.^120^ Incorporating MRI data enhanced the model's performance, yielding an excellent area-under-curve value of 1.00. The obstructive apnea index was identified as the key predictor, while heatmap visualisations highlighted anatomical regions associated with obstructive sleep apnea, including the nasopharynx, oropharynx, uvula, larynx, and epiglottis.^120^ An ML model was also able to automate the analysis of mandibular jaw movements for detecting sleep bruxism.^121^ AI integration with conventional diagnostic approaches has the potential to transform the management of sleep disorders, facilitating personalised treatment efficacy.

## Conclusions

AI is redefining dentistry by enhancing diagnostic accuracy, treatment planning, and patient care. AI-driven models, including machine learning, deep learning, and neural networks, have demonstrated high precision in radiographic analysis, disease detection, prosthodontic design, and orthodontic treatment planning. Indeed AI applications extend beyond clinical practice to foci such as forensic odontology and dental education, improving diagnostic efficacy and treatment planning. In addition, application of AI driven models in dental education should vastly improve the quality of both undergraduate and postgraduate education in future.

While AI continues to evolve, its successful integration into dentistry requires robust validation, interdisciplinary collaboration, and regulatory frameworks to ensure clinical reliability. Future advancements in AI will further refine treatment modalities, optimize workflows, and contribute to evidence-based, data-driven dentistry, ultimately improving patient outcomes and reshaping the future of dental care.

## Conflict of interest

All the authors declare no conflict of interest.
